# Citation practices in applied linguistics: a comparative study of Chinese expert and novice authors

**DOI:** 10.3389/fpsyg.2025.1515323

**Published:** 2025-04-01

**Authors:** Xue Gong, Ruoxi Liu, Chuanbo Ji

**Affiliations:** ^1^School of Humanities and Law, Hebei University of Technology, Tianjin, China; ^2^School of Chinese as a Second Language, Faculty of Humanities, Peking University, Beijing, China

**Keywords:** Chinese applied linguistics, citation practices, expert authors, novice authors, academic writing

## Abstract

Citation practices are crucial in academic discourse for both knowledge construction and interpersonal interaction. While prior research in academic English has explored citation practices among expert and novice authors, there is a notable gap in studies focusing on Chinese academic papers. Moreover, it remains uncertain whether insights from English-language corpora can be extrapolated to other linguistic contexts. This study presents a comparative analysis of citation practices among expert and novice authors within the field of Chinese Applied Linguistics. Utilizing a corpus of 715,000 Chinese words, we analyzed academic papers authored by both groups. Our findings reveal that citation practices between expert and novice authors are largely comparable. Specifically, integral citations were more prevalent than non-integral citations, with the cited authors predominantly occupying the subject position. In terms of citation form, the four types employed, in descending order of frequency, were summary, block quote, generalization, and quote. The analysis of reporting markers showed a predominance of discourse markers, followed by research markers, with cognitive markers being the least frequent. Notably, novice authors demonstrated certain deficiencies compared to their expert counterparts, including an overreliance on integral citations, a reduced use of generalization and block quote citations, and limited integration of information regarding reporting markers.

## 1 Introduction

Citations are a fundamental component of academic discourse, facilitating both the dissemination of ideas and the exchange of research findings within scholarly communities. The functions of citations can be categorized into three primary aspects: knowledge construction, intertextuality, and interpersonal interaction. At the level of knowledge construction, the generation of new knowledge relies on the integration of a shared disciplinary framework. Incorporating previous research findings is essential for constructing new insights, positioning citations as a vital tool for presenting and advancing scientific knowledge (Hyland, 1999). From an intertextual standpoint, citations extend the discourse beyond the immediate text, allowing for the integration of the current study with prior research in the field. This positions the research within the broader scholarly landscape (Hyland and Jiang, 2019). In terms of interpersonal interaction, citations facilitate engagement with two key groups. First, within the academic community, citations are used to assess existing research—through agreement, critique, or neutrality. These interactions foster dialogic relationships to advance disciplinary knowledge (Thompson and Ye, 1991). Moreover, to persuade readers effectively, citations of relevant studies are necessary to support the proposed arguments (Hyland, 1999; Hyland, 2010; Mansourizadeh and Ahmad, 2011; Chen and Zhang, 2017).

Citation practices have been a focal point across disciplines such as applied linguistics, sociology of knowledge, and information science (Swales, 1986; Bazerman, 1988; White, 2004). In applied linguistics, key areas of investigation include the forms and functions of citations (e.g., Harwood, 2009; Swales, 2014), interdisciplinary variations in citation practices (e.g., Hyland, 1999), differences between native and non-native English authors (e.g., Li and Zhang, 2021), and contrasts between expert and novice authors (e.g., Marti et al., 2019).

The majority of existing studies have focused on the examination of citations in academic English writing, with comparatively limited attention directed toward Chinese papers. Indeed, there are notable differences between citation practices in academic English and those in academic Chinese. For instance, while Arizavi and Choubsaz (2021) observed a greater prevalence of non-integral citations in academic English papers, Peng (2019) reported the opposite trend, namely that scholars who are trained in China exhibited a stronger tendency to use integral citations in their English papers. This also indicates that the influence of the mother tongue on citation practices is a factor that should not be overlooked. It is therefore necessary to analyse Chinese-language papers in order to gain new insights.

## 2 Literature review

### 2.1 Research on citation practice in academic writing

A substantial amount of research has been conducted on citation practices in academic writing. Some studies have examined the form and function of citation practices (Swales, 1986; Hyland, 1999; Thompson and Ye, 1991; Petrić, 2007; Mansourizadeh and Ahmad, 2011). Furthermore, studies have been conducted that have explored differences in citation practices in papers from different disciplines (Hyland, 1999; Hu and Wang, 2014; Wang and Hu, 2022), and differences among academic writers with different cultural backgrounds (Li, 2011; Cui and Cheng, 2014; Peng, 2019), and differences among writers with English as a second language versus native English speakers (Sun, 2009; Lou, 2011; Li, 2012; Shi, 2013; Lee et al., 2018; Li and Zhang, 2021), and differences in different genres (e.g., introductions, methodology, results, and discussion genres for empirical papers) (Martínez, 2008; Kwan and Chan, 2014; Zhang, 2022; Zhang, 2023).

In relation to the manner of citation practice, the extant research can be summarized as follows:

*Embedding method*: Swales (1986) classified citations as integral or non-integral, based on the position of the quoted person within or outside of the sentence. The academic community has endorsed this classification and it has been adopted by subsequent studies related to citation practices in academic writing (Hyland, 1999; Charles, 2006; Mansourizadeh and Ahmad, 2011; Samraj, 2013; Zhang, 2023; Mu, 2024). Furthermore, studies have been conducted that refined the categorization based on the syntactic position of the cited authors (Hyland, 1999; Thompson and Tribble, 2001). To illustrate this, Hyland (1999) categorized integral citations into the cited author as subject, non-subject, and situated in a noun phrase.

*Citation form*: Hyland (1999) divides the citation form into four categories: summary, generalization, quote, and block quote. Summary and generalization are indirect citations. Summary means that the material quoted is attributed to one source. Generalization means that the material is attributed to two or more sources. Quote and block quote are direct citations. Quote is a short direct quotation (three or more words). Block quote refers to extensive use of the original wording, set out as indented blocks. Borg (2000) and Petrić (2012) further classify direct quotations into three categories: quotation fragments (stretches of textual borrowing shorter than a T-unit), short quotations (T-units shorter than 40 words), extended quotations (quotations longer than 40 words).

*Reporting markers*: Thompson and Ye (1991) classified reporting markers into three categories, namely research markers (e.g., observe), cognitive markers (e.g., believe), and discourse markers (e.g., discuss). Hyland (1999) and Liu et al. (2021) followed this categorization.

### 2.2 Variation of citation practice by writer expertise

The differences in citation practices among writing groups with varying levels of expertise can be observed in five main ways.

First, writing groups with higher levels of expertise tend to have a higher citation density than those with lower levels of expertise. For example, Lombardi (2021) study demonstrated that high-level writers cite more frequently than low-level writers.

Second, with regard to the embedding method and citation form, writing groups with higher levels of expertise tend to employ a greater number of non-integral citations (Mansourizadeh and Ahmad, 2011; Ahn and Oh, 2024), and they tend to introduce shorter segments of source material (Lombardi, 2021).

Third, the use of reporting markers is more diverse in terms of the reporting verbs employed by writers with higher levels of expertise (Lombardi, 2021).

Fourth, writers with higher levels of expertise tend to evaluate the cited content and express their personal stance in their citation practices (Wette, 2018; Zhang, 2023). For example, Lombardi (2021) study demonstrated that high-level writers are more likely to attach personal evaluations to reporting markers than their less experienced counterparts. In contrast, low-level writers tend to avoid evaluative citations (Li and Zhang, 2021). Furthermore, doctoral students have been observed to utilize evaluative citations more frequently than their master’s counterparts (Zhao and Zhan, 2020).

Fifth, in terms of the function of citations, expert authors are more proficient in employing citations to serve their communicative purposes. For instance, doctoral students are more likely to cite sources than master’s students (Li and Zhang, 2021). It has been demonstrated that experts are more proficient in substantiating their personal discourses through the use of citations (Mansourizadeh and Ahmad, 2011; Mu, 2024). Additionally, experts are more inclined to engage in comparative analysis of research findings through the utilization of citations within the discussion section (Samraj, 2013). Furthermore, studies have examined the utilization of citations in research grant applications by novice authors, revealing that novice authors are in a transitional phase from student to researcher and have not yet developed the academic writing skills and competencies comparable to those of expert authors (Fazel and Shi, 2015).

A review of the existing research reveals three areas that warrant further investigation. First, the issue of expertise influencing citation practice is still in its infancy. The majority of articles were published subsequent to 2020, and the total number of articles is relatively limited. It is noteworthy that while existing academic writing textbooks address citation practices, they tend to focus on reminding writers to avoid plagiarism through proper citation, rather than on choosing the most appropriate form of citation for communicative purposes. This makes it challenging for novice writers to obtain effective guidance from these textbooks. Second, the extant studies utilize English papers as the corpus, with fewer studies focusing on citation practices in Chinese papers and an even smaller number of studies on the citation practices of novice Chinese academic writers. It has been demonstrated that there are differences between the academic citation practices of English and Chinese (Arizavi and Choubsaz, 2021; Peng, 2019). Consequently, it is necessary to re-examine the latter. Third, there are already established studies on the citation practices of novice authors, which compare dissertations with journal papers. For example, Li and Zhang (2021) and Ahn and Oh (2024) have already conducted such studies. However, it should be noted that dissertations and journal papers belong to two different genres. Therefore, further research is needed to explore whether citation practices can be compared across genres.

In light of the aforementioned background, this paper seeks to address two research questions by constructing a corpus of expert and novice academic Chinese journal papers:

1. Does the number of citations vary according to the level of expertise of the writers?
2. Does embedding method, citation form, and reporting markers of citations vary according to the level of expertise of the writer?

## 3 Methods

This study is based on a corpus of 190 journal papers, comprising a total of 715,000 words, drawn from two distinct writing groups with varying levels of expertise (experts/novices) in their respective fields. In consideration of the disciplinary variation in citation practices (Hyland, 1999), the corpus for this research is limited to that of applied linguistics. The rationale for selecting this discipline is based on the researchers’ familiarity with it, which ensures more reliable findings.

### 3.1 Data collection: the corpora

The corpora for this study are categorized into two segments: expert-authored papers and novice-authored papers.

The corpus of expert authors’ papers was created in the following manner. The citation analysis feature of China National Knowledge Infrastructure (CNKI) was employed to examine the most highly-cited authors in the field from five core journals between 2015 and 2020. The journals in question are *Chinese Teaching in the World*, *Language Teaching and Linguistic Studies*, *Applied Linguistics (Yuwan Wenzi Yingyong)*, *Chinese Language Learning*, and *Chinese Linguistics*. The 10 most prolific authors from each of the aforementioned journals were selected as potential experts. From this initial list, authors were further filtered based on their substantial individual publication record and significant recognition within the academic community. Ultimately, a total of 95 papers authored by 14 expert authors were chosen to establish the expert papers’ corpus, comprising 379,000 words.

The corpus of novice authors’ papers was created in the following manner. The data selected for the novice authors’ papers were sourced from the Graduate Forum organized by the School of Chinese as a Second Language at Peking University, spanning the years 2016–2019. The total number of papers included in the corpus is 158. The authors of these papers were all enrolled in master’s or doctoral programs and had prior experience with academic paper writing. However, as their academic writing skills were in the early developmental stage, they can be considered novices in academic writing. Papers authored by individuals with experience in publishing papers in core journals were excluded through manual screening. Furthermore, papers that did not comply with the standards required for journal publication or could not be converted to the requisite format were excluded. The screening process yielded 135 papers that were retained for further analysis. To ensure comparability with the expert journal papers corpus, 95 papers were randomly selected from the 135 retained papers for analysis, amounting to a total of 336,000 words. The conference papers have been incorporated into the CNKI database. Although they have not yet been published in academic journals, the objective of these papers is consistent with that of journal papers, namely to facilitate academic discourse and exchange between peers. Moreover, the length of both conference papers and journal papers is comparable. Therefore, in addition to the discrepancy in paper quality, they are, for the most part, comparable. However, they differ significantly from dissertations in terms of both the purpose of the writing and the length of the texts. Accordingly, for the purpose of citation analysis of academic papers, we treat journal papers and conference papers as essentially equivalent and utilize the term “journal papers” to refer to both in our discourse.

### 3.2 Citation identification and coding

The identification of citation examples involves a systematic three-step process. The first step employs the HanLP toolkit, developed in Python, to perform Named Entity Recognition (NER) within the corpus, identifying entities such as names of individuals, places, and organizations. The second step consists of filtering out statements that reference the names of individuals. In the third step, statements are manually reviewed to exclude those that do not pertain to cited literature. Subsequently, the remaining statements are categorized according to the analytical framework outlined below.

In order to validate the generalisability of the findings on academic English citation practices among authors with different levels of expertise, this paper employs a citation example analysis framework inspired by Hyland (1999) research. The framework enables a comparison of the quantity of citations present in the papers of expert and novice authors. Furthermore, the study examines the citation practice of the two groups from three dimensions: embedding method, citation form, and the use of reporting markers.

Embedding method is classified into two categories: non-integral, as exemplified by (1), and integral. Integral can be divided into three categories depending on the syntactic position of the cited authors. The first category comprises instances where the cited author is the subject of the sentence, as illustrated in example (2). The second category encompasses cases where the cited author is not the subject but appears as an additional constituent, as exemplified in example (3). The third category includes instances where the cited author is in a noun phrase, as demonstrated in example (4).

1. The integration of Chinese culture teaching with Chinese language instruction has always been one of the important research topics in the field of Chinese language teaching (Lu and Ma, 2016).
  中国文化教学与汉语语言教学的结合一直是汉语教学领域研究的重要课题之一(陆俭明, 马真2016).
2. Qiang (2010) distinguished between the topic marker “~嘛” and the modal particle “~嘛,” and described their process of grammaticalization.
  强星娜(2010)则区分了话题标记的“~嘛”和语气词的“~嘛”, 并描写了它们语法化的过程。
3. As Mr. Lu pointed out, language teaching materials should not be confined to the systematic nature of linguistic knowledge when dealing with language materials…
  正如鲁健骥先生所说, 语言教材在处理语言材料的时候, 不应拘泥于语言知识的系统性…
4. Among them, Sally’s (2007) five insightful suggestions are as follows: first…
  其中Sally (2007)提出的5条建议颇有见地…

Citation form is divided into four categories: block quote citation, which means that the quoted text is longer than or equal to 1 T unit, as illustrated in example (5); quote citation, which means that the original text is quoted as a word or phrase, as illustrated in example (6); summary citation, which means that the quoted text is a summary of one piece of literature, as illustrated in example (7); and generalization citation, which means that the quoted text is a summary of several pieces of literature, as illustrated in example (8). The above citation styles actually reflect the degree of integration of the original text by the author. Among them, block citation has the lowest level of integration of the original text, and the other three citation styles have increasing levels of integration, in that order.

5. The Ministry of Education’s Department of Teacher Education… defines it as “the continuous development process of teachers as individual professionals, involving their continuous acquisition of new knowledge and enhancement of professional capabilities. To become a mature professional, teachers need to expand the depth of their profession and improve their professional level through continuous learning and exploration, thus achieving a state of professional maturity.” (Wang, 2015)
  教育部师范教育司…, 将…界定为“教师个体专业不断发展的历程, 是教师不断接受新知识, 增长专业能力的过程。教师要成为一个成熟的专业人员, 需要通过不断的学习与探究历程来拓展其专业内涵, 提高专业水平, 从而达到专业成熟的境界” (王添淼 2015).
6. To eliminate the interference caused by relevant projects in language learning, George (1972) proposed an error prevention strategy called “orderliness of input,” suggesting that…
  为了消除相关项目给语言学习带来的干扰, George (1972) 提出一种称作“有序输入” (orderliness of input) 的错误预防策略, 认为…
7. For example, “没” (méi) evolved from a verb to an adverb and gradually transformed into a subjectively diminishing marker due to the constraints of subjective expression (Zhang, 2006).
  比如“没”从动词虚化为副词, 由于受到主观表达的制约逐渐转化成为主观减量标记(张谊生 2006).
8. The publication of the first set of Chinese textbooks for foreign language learners, “Chinese Textbooks,” in 1958 laid the foundation for… (Ke Bide, 1990; Li Quan and Jin Yunzhen, 2008, etc.)
  1958年第一套对外汉语教材《汉语教科书》出版, 奠定了… (柯彼德 1990; 李泉, 金允贞 2008等) (徐晶凝 2016).

The selection of reporting markers reflects the rhetorical competence evident in academic writing. By selecting appropriate language forms and establishing intertextual relationships with external content, the writer is able to achieve the communicative purposes within the discourse.

The classification of reporting markers can be divided into three categories, depending on the criteria used for differentiation: research markers, cognitive markers, and discourse markers. Research markers are primarily associated with research acts and can be further classified into two subcategories: those pertaining to the research process and those pertaining to the results of the research. Those markers that refer to the research process, such as “examined” and “counted,” etc., and those that refer to the results of the research, such as “found” and “constructed,” etc. Cognitive markers, which mainly refer to cognitive processes, e.g., “concerned” and “speculated,” etc. Discurse markers, which refer mainly to speech acts such as “pointing out,” “elaborating,” etc.

According to Liu et al. (2021) and the corpus, the structural form of reporting markers in Chinese academic papers is very flexible, so we also examine the differences in the structural form of reporting markers between expert and novice papers. The structural form can be classified into four categories. The first category comprises independent verbs or independent verbs with a tense component, which are abbreviated as “v + *le/guo*.” Examples of this category include “propose,” “proposed.” The second category is a prepositional phrase, which is abbreviated as “pre + v.” An example of this category is “*dui*…*jinxing*…*yanjiu*.” The third category comprises reporting verbs situated in relational clauses, which is abbreviated as “…v *de* n.” The fourth category encompasses the reporting verbs occupying the central clause position of a modifier-head structure, which is shortened to “…*de* v.”

In order to provide a more comprehensive overview of the analytical framework employed in this study, we have provided a summary of the aforementioned three categories in tabular form (see Table 1).

**Table 1:** Citation practice analysis framework.

Embedding method	Non-integral, e.g., (Hyland, 1999)		
	Integral	Subject. As a subject, e.g., “Hyland (1999) argues that…”	
		Noun-phrase. The cited author is located in a noun phrase and the noun phrase serves as a necessary syntactic component, e.g., “Hyland (1999) study found that…”	
		Adjunct. The cited author is located in an adjunct phrase, e.g., “According to Hyland (1999)…”	
Citation form	Quote, citing only a word or phrase from the original text		
	Block quotes, e.g., Hyland (1999) suggests that “…”		
	Summary, e.g., Hyland (1999)		
	Generalization, e.g., “Hyland (1999) and Jiang, (2005)”		
Reporting markers	According to the concept of righteousness, divided into three categories	Research markers	Referring to the research process, e.g., “observe”
			Referring to the results of research, e.g., “find”
		Cognitive markers, e.g., “notice”	
		Discourse markers, e.g., “point out”	
	According to the structural form, divided into four categories	Reporting verbs alone or reporting verbs with an additional tense element, e.g., “v + *le/guo*”	
		Prepositional phrase, e.g., “*dui*…*jinxing le*…v”	
		Reporting verbs are located in relational clauses, such as “…v *de* n”	
		Reporting verbs are located in the central clause position of a modifier-head structure, e.g., “…*de* v”	

## 4 Results and discussion

### 4.1 Overall comparisons of citation practices across corpora

Table 2 presents the citation counts, average citations per paper, and relative citation rates for authors with varying degrees of expertise. The data indicate that expert authors demonstrate higher average citation counts and citations per thousand words compared to novice authors. However, the difference between the two groups is not statistically significant (*χ*^2^ = 0.92, *p* > 0.05). This finding aligns with previous research in academic English, which suggests that papers authored by individuals with higher levels of expertise tend to exhibit relatively higher citation rates (Mansourizadeh and Ahmad, 2011). Nevertheless, similar to the current study, the discrepancy between the two groups remains statistically insignificant (Li and Zhang, 2021).

**Table 2:** Comparison of the number of citations by expert and novice authors.

Author Type	Number of citations	Average citations per paper	Relative citations (per 1,000 words)
Expert author	905	9.53	2.39
Novice author	765	8.05	2.28

The lower number of citations in novice authors’ papers may be attributed to a lesser degree of intertextuality awareness in this group. McCulloch (2013) conducted an exploratory analysis of the process undertaken by two master’s degree students from reading the material to writing a course paper, with a particular focus on the manner in which the authors utilized the source material to complete the paper. The study revealed that the level of intertextuality awareness exhibited by the authors varied considerably. Some of the authors demonstrated a higher degree of intertextuality awareness than others. This manifested in two ways. Initially, the authors demonstrated an active engagement with the source materials, extracting and adapting the information therein to express their own viewpoints. Secondly, they exhibited the ability to make connections between multiple source materials, extracting and utilizing the information after a critical comparison and reflection. In conclusion, authors with a high sense of intertextuality will consciously reshape information from source materials to apply it to their writing, and will actively expand and compare related materials for critical selection. Both of these behaviors can result in an increased number of discourse citations. It can therefore be surmised that the paucity of citations in the papers of novice authors is at least partly attributable to their limited awareness of intertextuality.

### 4.2 A comparison of expert and novice author citation practices

The number of citations in the papers of expert and novice authors is not significantly different. Nevertheless, this does not necessarily indicate that there are no discernible differences in the citation practices observed in Chinese academic papers between the two groups. The subsequent analysis will undertake a comprehensive comparative examination based on the framework presented in Table 1.

#### 4.2.1 Embedding method

A notable discrepancy was observed in the selection of the embedding method between the two author groups (see Table 3). Expert authors are more likely to utilize non-integral citations in comparison to their novice counterparts. As illustrated in Table 3, the proportion of non-integral citations among expert authors is 45.75%, whereas the corresponding figure for novice authors is 35.03%. This difference is statistically significant. This result is consistent with the findings of Mansourizadeh and Ahmad (2011), indicating that the observed effect of expertise on the choice of embedding style is generalizable across different linguistic contexts. Furthermore, academic Chinese exhibits distinctive characteristics with regard to the embedding method in comparison to English. In Chinese papers, there is a greater tendency toward integral than non-integral, whereas in English papers, the opposite is true.

**Table 3:** Statistics on different types of author embedding methods.

Author Type	Non-Integral		Percentage	Integral		Percentage
	Raw numbers	Per 1,000 words		Raw numbers	Per 1,000 words	
Expert author	414	1.09	45.75%	491	1.3	54.25%
Novice author	268	0.8	35.03%	497	1.48	64.97%
Chi-square	*χ*^2^ = 15.98, *p* < 0.05			*χ*^2^ = 4.19, *p* < 0.05		

There are three advantages to using non-integral citations as opposed to integrated ones. Primarily, situating the cited source outside of the sentence serves to accentuate the information contained within the citation, thereby facilitating a more objective presentation. Secondly, this approach enables authors to integrate the cited information seamlessly into their own viewpoint, thus making it an integral part of their argument. Thirdly, the use of non-integral citations ensures coherence within the discourse, preventing interruptions in the process of argumentation. These advantages of non-integral citations assist authors in developing their academic identities. In particular, the objective of introducing cited information is to construct the author’s viewpoint, and non-integral citations are an effective means of achieving this goal. Authors construct their academic identities by forming their own perspectives based on the cited information and expressing them within the academic discourse community (Ma and Qin, 2015). The restricted deployment of non-integral citations by novice authors suggests a lack of awareness of the potential to actively shape their academic identities. Rather than critically reflecting on established perspectives to form their own unique viewpoints, their aim in incorporating cited information is often to seek the “correct answer” or to present existing viewpoints.

In accordance with the established analytical framework, there are three distinct syntactic positions for the cited authors in integrated citations. A comparison of the results reveals significant similarities in the syntactic positions of cited authors between the two types of authors (see Table 4). First, no notable discrepancy was identified between the two groups of authors in the syntactic positions occupied by the cited authors as subjects or within noun phrases. However, a notable discrepancy is evident when the cited authors are situated within an adjunct phrase. This result differs from the findings of Mansourizadeh and Ahmad (2011), who observed a significant difference in the use of cited authors as subjects, with novice authors relying excessively on this structure (22.22%) compared to expert authors (6.56%). Second, both groups of authors demonstrate a markedly higher proclivity for utilizing cited authors as subjects, in comparison to the other two syntactic positions. The frequency of this pattern is markedly higher than the combined total of the other two patterns. This finding differs from that of Mansourizadeh and Ahmad (2011), in which the occurrence of cited authors as subjects was almost equal to the combined occurrence of the other two positions. Third, both groups of authors demonstrate a preference for utilizing cited authors in the following sequence: the preference for cited authors as subjects was observed to be the most frequent, followed by cited authors within an adjunct phrase and cited authors within a noun phrase. This observation is consistent with the conclusions of Arizavi and Choubsaz (2021), who conducted research on English-language academic journal papers and found that cited authors are most frequently placed as subjects, followed by prepositional phrases and noun phrases.

**Table 4:** Statistics on different types of author syntactic position.

Author type	Subject		Adjunct		Noun-phrase	
	Raw numbers	Per 1,000 words	Raw numbers	Per 1,000 words	Raw numbers	Per 1,000 words
Expert author	364	0.96	91	0.24	36	0.09
Novice author	368	1.09	111	0.33	18	0.05
Chi-square	*χ*^2^ = 3.01, *p* > 0.05		*χ*^2^ = 4.8, *p* < 0.05		*χ*^2^ = 3.52, *p* > 0.05	

The preceding analysis indicates that the syntactic positions of cited authors in Chinese journal papers differ significantly from those in English papers. Nevertheless, the overall distribution pattern remains consistent with that observed in English papers. These discrepancies may be attributed to the distinctive characteristics of Chinese academic papers. In contrast to English papers, Chinese papers tend to place the cited authors in the subject position with greater frequency. This form is more accessible for novice authors, which may contribute to the absence of a significant difference between the two groups. Conversely, English papers frequently employ nominalized phrases, which may prove more challenging for those with limited writing experience and/or non-native proficiency. Some studies have demonstrated that non-native speakers utilize a reduced number of nominalizations in their written work in comparison to native speakers (Tambul ElMalik and Nesi, 2008). Consequently, novice authors frequently utilize citations with the cited authors in the subject position. With regard to the similarities, the disciplinary nature of linguistics may be the reason. Despite the differences between the two language corpora, they both belong to the same field of linguistics. The syntactic positioning of the cited authors may serve to illustrate the disparate value placed upon them by the authors in question. The differences in ontology, epistemology and methodology among disciplines result in varying emphases being placed on the source and the knowledge it represents. For example, applied linguistics tends to emphasize the authority of the source, whereas clinical psychology prioritizes the expertise of the knowledge acquisition process (Hu and Liu, 2020). The findings of this study indicate that both English and Chinese papers tend to cite authors in prominent subject positions, which can be attributed to the disciplinary nature of linguistics.

#### 4.2.2 Citation form

A comparative analysis was conducted to examine the citation practices of expert and novice authors across four distinct categories. It was observed that, with the exception of quote, the two groups demonstrated notable discrepancies in their utilization of the remaining three citation forms, as illustrated in Table 5.

**Table 5:** Statistics on different types of author citation form.

Author type	Block quote		Quote		Summary		Generalization	
	Raw numbers	Per 1,000 words	Raw numbers	Per 1,000 words	Raw numbers	Per 1,000 words	Raw numbers	Per 1,000 words
Expert author	178	0.47	104	0.27	476	1.26	147	0.39
Novice author	94	0.28	77	0.23	509	1.51	85	0.25
Chi-square	*χ*^2^ = 16.43, *p* < 0.05		*χ*^2^ = 1.27, *p* > 0.05		*χ*^2^ = 8.45, *p* < 0.05		*χ*^2^ = 9.6, *p* < 0.05	

The discrepancies in the citation form between the two cohorts of authors can be encapsulated as follows: First, expert authors tend to employ a greater number of direct quotations in comparison to novice authors. This is demonstrated by the higher frequency of “block quote” and “quote” observed in the papers of expert authors in comparison to those of novice authors. This finding is consistent with the findings of Lombardi (2021), which also revealed an increase in the use of direct quotations with the writer’s level of expertise. Similarly, our study revealed that expert authors, who demonstrated greater proficiency, employed a greater number of direct citations than novice authors. The restricted deployment of direct quotations by novice authors indicates a diminished intertextual consciousness and affinity with source materials during the writing process. However, there is a discrepancy between the findings of our study and those of Lombardi (2021). While Lombardi (2021) observed a reduction in quotation length with increasing expertise levels, our study found that expert authors used longer “block quote” more frequently than novice authors did. We attribute this discrepancy to the differing nature of the corpora employed in each study. The papers of expert authors frequently comprise theoretical works that are heavily reliant on previous viewpoints. Consequently, it is imperative that they remain faithful to the original texts in order to guarantee the veracity of their arguments. Conversely, the papers of novice authors tend to comprise a greater proportion of content oriented toward application, which results in a lower incidence of opinion-based citations and a reduced necessity for extensive block quotations. Consequently, such citations are employed less frequently by novice authors.

A second distinction can be observed in the use of citations by expert and novice authors. Expert authors employ a greater number of generalization citations and a smaller number of summary citations compared to their novice counterparts. The utilization of generalization citations fulfils two distinct rhetorical functions. Primarily, it serves to enhance the credibility and authority of the content presented, thereby providing support for the author’s viewpoints or claims. This approach to citation enables authors to adapt the cited content in a flexible manner, thus enhancing the effectiveness of their argumentation and achieving the communicative goal of persuading readers (Hyland, 1999). Second, it establishes connections among numerous studies within the same field (Petrić, 2007), thereby demonstrating the author’s familiarity with the research domain and their ability to present themselves as an expert in academic writing. The restricted deployment of such citations by novice authors also suggests a deficiency in their intertextual awareness with regard to existing research, as well as a lack of awareness of the selection of citation approaches that may be employed in order to construct an academic expert identity.

#### 4.2.3 Reporting markers

A preliminary statistical analysis was conducted to examine the proportion of reporting markers utilized in citations and the frequency of high-frequency reporting verbs employed by the two groups of authors (see Table 6). It was observed that there were no significant differences in the frequency of reporting marker usage between the expert and novice authors.

**Table 6:** Percentage of reporting markers and high frequency reporting verbs.

Author type	Number of reporting markers		Percentage	High frequency reporting verbs (Frequency > 8)
	Raw numbers	Per 1,000 words		
Expert author	610	1.61	67.4%	point out (指出), think (认为), find (发现), say (说), propose (提出), study (研究), analyze (分析), explore (探讨), advocate (主张), examine (考察), classify (分为), 讨论 (discuss)
Novice author	566	1.68	73.99%	think (认为), propose (提出), point out (指出), analyze (分析), study (研究), examine (考察), mention (提到), explore (探讨), classify (分为), summarize (总结)
Chi-square	*χ*^2^ = 0.55, *p* > 0.05			

The use of reporting verbs indicates that both groups of authors frequently utilize a similar set of high-frequency reporting verbs. However, expert authors demonstrate a higher level of diversity in their use of reporting verbs compared to novice authors, as evidenced by two key aspects. First, expert authors demonstrate a greater diversity of reporting verb types, resulting in a higher Type-Token Ratio (TTR) of reporting verbs in their corpus compared to novice authors. In particular, the TTR value for reporting verbs in the corpus of expert authors is 0.21, while in the corpus of novice authors, it is 0.2. Second, with regard to the coverage of high-frequency reporting verbs, the corpus of expert authors demonstrates that the top 10 high-frequency reporting verbs account for 53.11% of the total occurrences, whereas in the corpus of novice authors, the top 10 high-frequency reporting verbs cover 61.13% of the total occurrences. This suggests that novice authors tend to focus on utilizing the 10 most prevalent reporting verbs, exhibiting a lesser degree of complexity and adaptability in their paraphrase verb usage compared to expert authors. These findings are consistent with those of Lombardi (2021), which revealed that high-level authors exhibited a more diverse range of reporting verbs in their writing.

By analyzing the use of reporting markers with varying referential content, it is possible to ascertain the authors’ preferences with regard to the selection of original material. The comprehensive statistical findings are presented in Table 7. The distribution of the three types of markers is consistent in the corpora of both groups of authors. Discourse markers are the most frequently used, followed by research markers, and cognitive markers are the least used. However, there is a discernible discrepancy in the usage pattern between novice and expert authors. The data indicates that novice authors tend to utilize research process markers with greater frequency, while employing research result markers with lesser frequency, in comparison to expert authors. Lombardi (2021) observed that high-level authors tend to utilize reporting verbs that reflect their current discursive actions, such as “argue,” to express their evaluation of the cited content. In comparison to research process reporting markers, research result markers are more likely to convey evaluative information. To illustrate, the research result-oriented reporting marker “证实 (confirm)” indicates the author’s affirmative evaluation of the cited content. The restricted deployment of research result reporting markers by novice authors indicates a deficiency in their ability to critically evaluate the cited information.

**Table 7:** Distribution of three types of reporting markers.

Author type	Research markers						Discourse markers		Cognitive markers	
	Research act		Research results		Total					
	Raw numbers	Per 1,000 words	Raw numbers	Per 1,000 words	Raw numbers	Per 1,000 words	Raw numbers	Per 1,000 words	Raw numbers	Per 1,000 words
Expert author	171	0.45	59	0.16	230	0.61	365	0.96	15	0.04
Novice author	211	0.63	24	0.07	235	0.7	315	0.94	16	0.05
Chi-square	*χ*^2^ = 10.07, *p* < 0.05		*χ*^2^ = 10.19, *p* < 0.05		*χ*^2^ = 2.19, *p* > 0.05		*χ*^2^ = 0.1, *p* > 0.05		*χ*^2^ = 0.11, *p* > 0.05	

From a structural form perspective, there are significant differences in the use of the four types of structural forms between expert and novice authors (see Table 8). In particular, expert authors tend to favor the use of “v + *le/guo*” and “…*de* v,” while novice authors tend to use “pre + v.” The “…*de* v” structure serves two functions. First, it nominalizes the research process, making the expression more formal in writing style, as seen in example (9) with the word “调查” (investigation). Secondly, this structure provides syntactic positions for multiple paraphrased content. In example (9), it introduces the research object with the preposition “对” (regarding), and in example (10), it incorporates the manner information related to the reporting verb “倡导” (advocate) with the term “大力” (vigorously).

9. Tao Hongyin’s investigation of Chinatowns in the United States found that “compared with Mandarin in Hong Kong, Taiwan, and Southeast Asia, North American Chinese is more like a great fusion of Chinese varieties, as its users consist of immigrants from these diverse regions within the Chinese cultural sphere” (Li Yuming, 2017).
  陶红印对美国唐人街的调查发现:“跟港台, 东南亚地区的华语相比, 北美汉语更像是一个汉语变体的大融合, 因为北美汉语使用者正是来自这些不同地区但同属中华文化圈的移民” (李宇明 2017).
10. Under the strong advocacy of Nattinger and De Carrico (1992), Lewis (1993, 1997), and others, the lexical approach, also known as “词汇法” in Chinese, has gradually become a influential teaching methodology.
  在 Nattinger and De Carrico (1992), Lewis(1993, 1997) 等的大力倡导下, 语块教学法 (lexical approach,或译作“词汇法”) 逐渐成为一种较有影响的教学法流派.

**Table 8:** Distribution of reporting markers’ structural form.

Author type	v + *le/guo*		pre + v		…*de* v		…v *de* n	
	Raw numbers	Per 1,000 words	Raw numbers	Per 1,000 words	Raw numbers	Per 1,000 words	Raw numbers	Per 1,000 words
Expert author	416	1.1	139	0.37	33	0.09	22	0.06
Novice author	302	0.9	244	0.73	5	0.01	15	0.04
Chi-square	*χ*^2^ = 6.86, *p* < 0.05		*χ*^2^ = 42.25, *p* < 0.05		*χ*^2^ = 16.15, *p* < 0.05		*χ*^2^ = 0.39, *p* > 0.05	

The second function of this structure is to encapsulate the reporting information, allowing great flexibility in syntactic positioning and facilitating subsequent comments or evaluations. In example (11), the encapsulated information appears in the subject position and the author provides an evaluation of it. In example (12), the encapsulated information is placed in the object position, explaining the concept of “句本位” (sentence-based perspective). Similarly, in example (13), it also occupies the object position, illustrating the basis of “教学呈现的先后顺序” (the order of instructional presentation), followed by further details of “主张” (proposition).

11. The analysis by Thomason is remarkably clear… (Zhang Bo, 2019)
  Thomason的分析非常清楚,… (张博 2019).
12. Meanwhile, “句本位” is the proposition employed by Li Jinxi to elucidate the fundamental ideas of grammar (Zhao Jinming, 2017).
  而“句本位”则是黎锦熙用以揭示语法基本思想的主张 (赵金铭 2017).
13. The sequence of presentation in teaching is based on Mr. Zhao Yuanren’s proposition, primarily considering the frequency of phrase and structure usage (Zhao Jinming, 2018).
  教学中呈现的先后顺序, 是依赵元任先生的主张, 主要考虑短语和结构的使用频率 (赵金铭 2018).

The “…*de* v” structure falls into the category of nominalization, which serves as a crucial “linguistic carrier” for conveying information in academic discourse (Gui, 2014, p. 51). The prevalence of this structure among expert authors indicates their ability to use language structures that are in line with academic discourse to reporting others’ research and ultimately achieve their communicative goals.

There are differences in the temporal components attached to the reporting verbs used by the two groups of authors (see Table 9). Expert authors use “v + *guo*” more frequently and “v + *le*” less frequently compared to novice authors. Upon analyzing the corpus, we found that “进行了” (conducted) and “进行过” (have conducted) often alternate. To explore the differences in their usage, this study utilized Antconc 4.2.4 to examine high-strength collocates within the 8-word range to the right of both expressions. In the expert authors’ corpus, the top 3 high-strength collocates for “进行了” are “统计” (count), “研究” (study), and “分析” (analyze), all of which belong to research reporting markers. On the other hand, the top 3 high-strength collocates for “进行过” are “论述” (discuss), “探讨” (explore), and “讨论” (discuss), which are all discourse reporting markers. The advantage of the “进行过 + discourse reporting marker” combination lies in its ability to provide an overall report of previous research, including but not limited to the research process, with a stronger focus on the research results. This higher level of abstraction in the overall reporting allows the author to omit unnecessary reporting information, enabling them to emphasize their evaluation of previous research findings effectively.

**Table 9:** Tense and aspect in reporting markers.

Author type	v + *le*		v + *guo*	
	Raw numbers	Per 1,000 words	Raw numbers	Per 1,000 words
Expert author	60	0.16	30	0.08
Novice author	139	0.41	8	0.02
Chi-square	*χ*^2^ = 40.79, *p* < 0.05		*χ*^2^ = 9.26, *p* < 0.05	

## 5 Conclusion

Citations are an indispensable element of academic discourse, serving a pivotal function in the construction of knowledge, the interpretation of texts, and the dynamics of interpersonal communication. A substantial body of research on citations has been conducted, yielding a plethora of findings pertaining to various aspects of citations, including their forms, functions, and patterns across diverse contexts. Furthermore, differences in citation practices due to varying levels of expertise have been well-established in the field of academic English research. Nevertheless, it remains unclear whether the conclusions drawn from academic English corpora can be generalized to academic Chinese corpora. This study, which employed a self-constructed small-scale corpus, compared the similarities and differences in citation practices between expert and novice authors. The findings yielded three main conclusions:

First, it can be concluded that the findings derived from the analysis of academic English corpora can be largely extrapolated to academic Chinese corpora. This suggests that the impact of expertise on citation practices in academic journal papers is a cross-linguistic phenomenon. This study demonstrates that in academic Chinese writing, expert authors and novice authors exhibit comparable differences in citation density, embedding methods, citation forms, and reporting markers, as observed in academic English. For instance, expert authors are more likely to utilize non-integral citations and direct quotations, employ a diverse array of reporting markers and exhibit discernible proclivities in the utilization of evaluative reporting markers.

Moreover, academic Chinese exhibits distinctive features that set it apart from academic English. In academic Chinese papers, authors demonstrate a greater proclivity for employing the form of situating citees in subject position in comparison to their counterparts in the field of academic English. This form is readily comprehensible, resulting in no notable discrepancies in its utilization between expert and novice authors. Conversely, academic English writing tends to favor nominalized phrases, with the cited author situated within a noun phrase. This structure can prove challenging for novice authors to master, leading to an overreliance on the citees as subject form and resulting in significant usage differences between the two groups in this regard.

Ultimately, the discrepancies in citation practices between the two cohorts of authors can be attributed to their comparatively weaker intertextual awareness and less pronounced sense of developing an academic writing expert identity. In particular, novice authors tend to introduce cited information with the objective of identifying the “correct answer,” rather than engaging in a critical integration of disparate pieces of information and establishing intertextual relationships between the current discourse and multiple source materials. This approach fails to demonstrate their expertise or construct an expert identity in academic writing. The latter is achieved through synthesizing various sources, forming their own academic perspectives and highlighting their professional knowledge.

Two limitations remain in this study. First, the analytical framework addresses the form of citation, but not the function of citation. A combination of formal and functional analyses would have enabled the formulation of more operational pedagogical suggestions and provided novice authors with a clearer understanding of the appropriate citation forms for fulfilling communicative purposes. Second, the analysis is confined to the textual corpus; however, if interviews with novice and expert authors were to be incorporated, the motivations behind the observed differences in citation use between the two groups could be subjected to more rigorous analysis, thereby enhancing the reliability of the conclusions drawn.
